# Real-Time 3D Object Detection and SLAM Fusion in a Low-Cost LiDAR Test Vehicle Setup

**DOI:** 10.3390/s21248381

**Published:** 2021-12-15

**Authors:** Duarte Fernandes, Tiago Afonso, Pedro Girão, Dibet Gonzalez, António Silva, Rafael Névoa, Paulo Novais, João Monteiro, Pedro Melo-Pinto

**Affiliations:** 1 Algoritmi Centre, University of Minho, 4800-058 Guimarães, Portugal; dduartefernandes@gmail.com (D.F.); asilva@algoritmi.uminho.pt (A.S.); rafael.accn@gmail.com (R.N.); pjon@di.uminho.pt (P.N.); joao.monteiro@dei.uminho.pt (J.M.); pedro.melo@algoritmi.uminho.pt (P.M.-P.); 2 Bosch Company, 4700-113 Braga, Portugal; tiago.afonso@pt.bosch.com (T.A.); pedro.girao@pt.bosch.com (P.G.); 3 Computer Graphics Center, University of Minho, 4800-058 Guimarães, Portugal; 4 Department of Engineering, University of Trás-os-Montes and Alto Douro, 5000-801 Vila Real, Portugal

**Keywords:** autonomous driving, deep learning methods, LiDAR scanners, 3D object detection, onboard inference, SLAM, vehicles setup

## Abstract

Recently released research about deep learning applications related to perception for autonomous driving focuses heavily on the usage of LiDAR point cloud data as input for the neural networks, highlighting the importance of LiDAR technology in the field of Autonomous Driving (AD). In this sense, a great percentage of the vehicle platforms used to create the datasets released for the development of these neural networks, as well as some AD commercial solutions available on the market, heavily invest in an array of sensors, including a large number of sensors as well as several sensor modalities. However, these costs create a barrier to entry for low-cost solutions for the performance of critical perception tasks such as Object Detection and SLAM. This paper explores current vehicle platforms and proposes a low-cost, LiDAR-based test vehicle platform capable of running critical perception tasks (Object Detection and SLAM) in real time. Additionally, we propose the creation of a deep learning-based inference model for Object Detection deployed in a resource-constrained device, as well as a graph-based SLAM implementation, providing important considerations, explored while taking into account the real-time processing requirement and presenting relevant results demonstrating the usability of the developed work in the context of the proposed low-cost platform.

## 1. Introduction

Autonomous vehicle technology is making a prominent appearance in our society in the form of advanced driver assistance systems (ADAS) in both research and commercial vehicles. These technologies aim to reduce the amount and severity of accidents (these vehicles should be able to reduce 75–80% of current traffic fatalities [1], increase mobility for people with disabilities [2] and the elderly, reduce emissions, and use infrastructure more efficiently. In a simple way, autonomous vehicle navigation can be understood as encompassing five main components (Perception, Localization and Mapping, Path Planning, Decision Making, and Vehicle Control). Perception is all about forming an accurate and detailed environmental model of what is around you. The data used come from different types of sensors such as cameras (monocular or stereo), RADAR (RAdio Detection And Ranging), and LiDAR (Light Detection and Ranging) sensors. The LiDAR sensor is becoming a key element in self-driving cars, mainly because of its long-range scanning abilities (360° scanning, its high resolution provides full 5D information (X, Y, and Z coordinates, reflectivity, and time)) and the good performance under different lighting conditions (night or day) due to its light sources, i.e., lasers. Unlike cameras, LiDAR is not blinded when pointed in the direction of the sun and at night, LiDAR has a very high performance [3]. Depending on the LiDAR sensor, the data per point can be range, intensity, reflectivity, ambient, angle, and timestamp. However, the LiDAR sensor has some disadvantages, namely, it is still expensive technology compared to other solutions, it is affected by some specific atmospheric weather conditions, as in the case of fog and smoke presence. Research work [4] has shown that recent LiDAR sensors (higher angle resolution) perform well in rainy conditions. However, there are still several weather conditions requiring analysis; freezing rain, sticky snow, as well the temperature LiDAR range are some examples of variables that have not been considered in these LiDAR benchmarks studies. Moreover, sensors cameras, which might be found in other vehicles or traffic cameras, are susceptible to laser damage [5]. All these issues might hamper perception systems based on LiDAR to cover all scenarios.

Information provided by LiDAR, in the form of a 3D point cloud, has been processed for providing two fundamental components to reach the highest levels for autonomous driving (levels four and five), namely, Perception and Localization/Mapping. Perception is here described as the autonomous vehicle ability to detect objects in the surrounding environment in a real-time manner. However, this is a very challenging task due to the sparse and unstructured nature of the high dimensional data contained in point clouds, the number of points in a point cloud (which typically comprises more than 120,000 points [4]) and the limitations in computation power and power supply as expected in real-case applications, where vehicle setups do not contain a GPU-based server. Turning the task of accomplishing a perception algorithm able to meet the requirements imposed by the application, where it is expected for solutions to deliver outputs in a real-time manner (often, the target inference time is set as 10 Hz) [6], almost impractical. Autonomous perception has been focusing on learned features following the steps of deep learning object detection and classification algorithms based on Convolutions Neural Networks (CNNs) that have proven to be competitive with increased performance [6]. The inference time performance of such solutions addressed in the literature on public benchmarks is higher or near to the maximum acceptable value, even though solutions are deployed in powerful servers. Hence, the impossibility of directly deploying the computationally intensive CNNs on the resource-constrained processing units equipped in the vehicle setup is foreseen, as performance degradation is expected.

Localization/Mapping refers to the ability to determine the global and local location of the ego vehicle and map the environment/surroundings from sensor data and other perception outputs [7]. This component includes the problem of determining the pose of the ego vehicle and measuring its own motion. Due to the difficulty of determining the exact pose (position and orientation) of the vehicle, the localization problem is often formulated as a pose estimation problem [7]. Currently, the process of map building and localization, while driving, is known as SLAM (Simultaneous Localization and Mapping) and is an important process in the autonomous vehicles field. Visual SLAM algorithms are a subgenre of these methods, which only use visual information. For this process, LiDAR devices are more convenient to scan outside environments. LiDAR SLAM (SLAM supported by LiDAR) typically includes tasks such as odometry, scene recognition, loop closing, and re-localization to improve the map quality and keep the mapping precision [8].

This research project aims at developing and integrating a real-time 3D object detection and a SLAM algorithm, both based on LiDAR 3D measurements (where higher-level features, rather than the direct use of the point cloud, are followed) into a vehicle setup. This paper explores current vehicle platforms and proposes a low-cost, LiDAR-based test vehicle platform capable of running critical perception tasks (Object Detection and SLAM) in real time.

To the best of the authors’ knowledge, this is the first academic attempt of on-board inference focusing on the deep learning algorithms processing point clouds for driverless vehicles application. Here, we demonstrate how the computation-intensive inference algorithms can be deployed for resource-constrained edge devices. The proposed SLAM comprises several features that mitigate the error drift, resulting in an improved ability for mapping and locating relevant objects, while overcoming the main limitation of such algorithms, the inability to close loops. Moreover, both 3D object detection and SLAM algorithms are both integrated into the same test vehicle, continuously processing the same data, i.e., point cloud provided by the scanner LiDAR equipped in the test vehicle. This setup is a low-cost version, as it does not follow a multi-model approach as is typically seen in the literature, where vehicles are designed for dataset generation.

The paper is organized as follows: Section 2 describes related work regarding systems for 3D object detection, SLAM, and vehicles setup used in the literature for the acquisition of points. Section 3 addresses the architecture of the proposed test vehicles, where all the components adopted are discussed, a five-step methodology used to select, train, and fine-tune a deep learning model for deployment in a hardware device is discussed, and the overview of the architecture of the proposed SLAM algorithm is presented. Section 4 presents benchmarks of the floating-point and integer versions of the models previously presented in Section 3. In Section 4, the comparison of the quantitative test of the floating-point and integer versions of each model configuration are performed, and the performance results of the SLAM are presented and discussed. Finally, Section 5 presents the conclusions of the research and development carried out throughout this project.

## 2. Related Work

### 2.1. Object Detection

Over the last few years, the number of 3D Object Detection (OD) methods discussed in the literature has increased exponentially. These detectors targeting perception applications for driverless vehicles are categorized into single-stage architectures, e.g., models SECOND [9], PointPillars [10], and Fusion of Fusion Net [11], and dual-stage solutions [6], such as Fast Point-RCNN [12], Patch Refinement [13], and Part-A2-anchor [14]. The former architecture integrates stages as follows: (1) data preprocessing, (2) features extraction CNN-based backbone, and (3) multi-head detection head, as a set of connected layers to directly perform object detection and classification. On the other hand, dual-stage approaches add a new stage between stage (2) and (3) to generate a set of region proposals, called intermediate proposals, using a proposal generator, such as Region Proposal Network (RPN). These proposals are further refined by subjecting their content to additional feature extraction layers, which are then inputted into stage (3). Single-stage detectors are known for being more time-efficient but offer lower precision compared to dual-stage detectors. For instance, SECOND and Patch Refinement achieve a precision of 84 and 89%, and an inference time of 40 and 150 ms, respectively, for car detection in easy difficulty detection mode for the KITTI benchmark [15].

Resource-constrained Edge-devices, such as GPUs, FPGAs, and ASICs, have limited computation power and memory but can be easily integrated into the vehicle setup due to their low form-factor and low power consumption. Dedicated hardware accelerators have gained prominence due to their robustness, flexibility (unlike ASICs) and performance as they achieve a higher performance per watt than microcontrollers or GPUs [16]. Meeting applications’ requirements is a challenging task for edge devices. Inspired by research work [17], which consisted of the first hardware-software codesign resulting in a parameterized and runtime configurable FPGA hardware architecture compatible with several networks, several frameworks, such as HADDOC2 [18], DNNWEAVER [19], Hls4ml [20], and Vitis AI [21] have emerged, focusing not only on compiling the model for the target platform but also on compressing the network to reduce the memory footprint and inference time. Optimization techniques such as quantization, pruning, and code optimizations are often adopted. The differences between the frameworks are in the supported (1) input model format, (2) deep learning operators, (3) optimization techniques, and (4) platform devices. Vitis AI is the most complete framework, as is the only one that supports the widely adopted machine learning libraries (Caffe, TensorFlow, and Pytorch) for a large set of platforms from Xilinx (but does not support Intel platforms such as HADDOC2 and DNNWEAVER). This framework supports ample basic functions of deep learning and not only specific functions or CNNs [16]. However, unlike HADDOC2 and Hls4ml, user licenses are required to fully utilize the framework capabilities.

### 2.2. SLAM

Since the emergence of SLAM algorithms, specifically graph-based algorithms, the following two tasks stand out in the whole process: Odometry and graph optimization. Odometry consists of aligning successive LiDAR scans using a point cloud registration technique. By successively composing these transformations, each point cloud is transformed back to the reference frame of the first point cloud. Finally, the map can be gendered by combining all the transformed point clouds. Algorithms such as Normal Distributions Transform (NDT) [22,23] and Generalized Iterative Closest Point (GICP) are the most adopted methods. However, both algorithms are affected by the nearest neighbors searching on a dense points cloud, when is necessary to run applications in real-time. Algorithms in the literature, such as [3,24], resort to parallelization technics to overcome this limitation. Although odometry solutions create consistent maps, they are only suitable for mapping small areas, as an error drift is expected for longer sequences. To overpass this obstacle, the graph-optimization approach has been adopted. Here, a graph is incrementally created, where the nodes and the edges represent the vehicle absolute poses and the relative poses, respectively.

Well-known methods, such as Levenberg–Marquardt (LM), Gauss–Newton, or variants from gradient descent, have been adopted in many applications with acceptable results. However, to reach values in accuracy and efficiency with use-value, hard efforts, and advanced mathematical techniques are necessary. Furthermore, correct parameterization is necessary.

The open-source C++ library general graph optimization (g2o) [25] is an instance that implements standard methods such as Gauss–Newton and Levenberg–Marquardt (LM) for the optimization of nonlinear least-squares problems. To enhance its performance, this library exploits the sparse connectivity of the graph by adopting and using advanced methods to solve sparse linear systems. Moreover, it takes advantage of modern processors such as SIMD instructions and cache usage optimization techniques.

However, the efficiency of the graph optimization (as in g2o) decreases due to the increase in its size (more nodes and edges), which increases with the trajectory. This problem can be dimmed using graph pruned technics [26].

On the other hand, the initialization of the graph far away from the global minimum might drastically affect its efficiency. The values from odometry are used frequently to initialize the graph. However, it is known that it is not a convenient choice because of error accumulation with the trajectory. Adding different kinds of edges constraints helps to mitigate the odometry drift drawback, then improving the effectiveness of the graph optimization. Loops and floor plane detection are two community constraints used. NDT is often adopted for feature extraction on the loop detection [27], while RANSAC is the preferable algorithm for the floor detection task [28]. On the other hand, the graph optimization accuracy can be affected by a trajectory without loops. Furthermore, when constraining edges are added to the graph, a registration process is performed among the edge points.

Finally, the registration between point clouds, loop detection, and graph optimization is the most nuclear process, affecting the tradeoff between system efficiency and accuracy. The registration between point clouds has a linear efficiency, i.e., it is the same throughout the trajectory of the vehicle; its efficiency depends on the number of points used in the registration between the current and the previous point cloud. However, the efficiency of the graph optimization decreases due to the increase in its size (more nodes and edges), which increases with the trajectory. Summing up, SLAM task performance is tight bounded to the parameter settings in its main processes.

### 2.3. Test Vehicles

Perception and Localization/Mapping are fundamental components to reach the highest levels for Autonomous Driving (AD). Sensors providing support to these components fit in a wide array of families, including, but not limited to, (1) cameras, (2) LiDAR, (3) Radar (Radio Detection and Ranging) for Perception, (4) GNSS (Global Navigation Satellite Systems), and (5) IMU and odometers for localization/mapping:

1. Camera technology is currently the most widely considered for autonomous vehicles. Cameras capture images with detailed texture information; however, they are prone to weather conditions and changes in illumination. Thermal cameras are more robust to different weather conditions and illuminations due to reading the heat signatures of target objects in the scene, and operate on longer infrared (IR) wavelength regions, thus not seeing reflected light;
2. LiDAR sensors provide 3D point clouds that accurately represent depth data relating to surrounding environments structures and objects. Comparatively to passive cameras, LiDAR sensors are considered active sensors as they emit laser beams at a determined frequency, relying on the received reflections of the emitted beams to infer data such as distance and signal intensities to characterize reflecting surfaces;
3. RADAR sensors emit waves in radio frequency, which are reflected by surrounding structures and objects. Unlike cameras, they have greater robustness to illuminance and weather variations, but offer more challenges for perception application such as OD due to the lower resolution of the acquired data;
4. GNSS use a global satellite system, such as GPS and GLONASS, to provide accurate data related to the vehicle’s absolute position in the world;
5. IMU and odometers measure the vehicle’s acceleration/rotation data and odometry data, respectively. Unlike the previously considered sensor families, the data obtained from these sensors are internal to the vehicle, and when used with other sensors, can greatly help with estimating an accurate localization of the vehicle.

The currently used sensors in the AD area hint at the existing benefits of using a multi-modality approach. Data collection for the creation of datasets is an example of an application that greatly benefits from sensor fusion. However, as one starts adding sensor modalities and the number of sensors to a vehicle, aiming to increase the coverage of the vehicle’s surroundings, the cost of building such an infrastructure raises sharply, both related to the price of the sensors themselves but also to the inherent costs of retrofitting a vehicle with the sensor setup and legal procedures for the test vehicle to be able to drive and acquire data from open public roads.

Many AD test vehicles are fitted with an array of sensors as described. An overview of the existing vehicle platforms and sensor setups is presented in Table 1. The DARPA Urban Challenge [29] showcased one of the first autonomous vehicles, Boss, which mostly used a combination of a camera, RADAR, and LiDAR sensors to achieve first place in the competition.

Additionally, in the same year, Montemerlo et al. [30] presented Junior, a robotic vehicle also capable of navigating urban environments autonomously, achieving second place in the same competition. In this challenge, the Boss vehicle platform had roughly twice the amount of LiDAR sensors and also used two camera sensors. Google’s Waymo [34] also uses LiDAR, camera, and RADAR sensors for perception tasks, and also includes an IMU, GPS, and audio capturing sensors such as microphones. Additionally, many vehicles have been used as mobile platforms for dataset creation, which pushes AD research forward. An extensive survey on the existing multi-modal datasets and corresponding data vehicles used can be found in [34]. Two of the most used datasets in the field of research are KITTI and nuScenes. KITTI is a multi-sensor moving platform aimed at enabling research on stereo, optical flow, visual odometry/SLAM, 3D object detection, and 3D tracking tasks [3]. The vehicle platform features stereo camera systems, a LiDAR sensor and an OXTS RT 3003 localization system, which combines GPS, GLONASS, an IMU, and RTK correction signals. According to the authors, to compensate ego motion in the 3D laser measurements, they used the position information from the GPS/IMU system. nuScenes is a public large-scale dataset for AD developed by Aptiv Autonomous Mobility and is a multi-sensor/multimodal moving platform that can be used for multiple computer vision tasks, such as object detection and tracking. As with KITTI, the vehicle platform features an array of sensors including RGB cameras, LiDAR, and RADAR sensors.

From the presented vehicle platforms and sensor setups, some key takeaways can be extracted: most solutions aim at dealing with multiple complex tasks such as perception tasks for AD scenarios, requiring costly hardware and configurations for the performance of these tasks, or dataset creation, where such vehicle platforms often include a multitude of sensor modalities and a large number of sensors overall to try and cover a high number of scenarios in different conditions (weather, buildings, roads, etc.). This, in turn, means spending a considerable amount of funds to fully equip these vehicles, including data processing platforms such as costly network configurations and computer hardware. In this sense, none of the above-mentioned works, and virtually no related works in the literature, to the best of the authors’ knowledge, aim at providing a low-cost infrastructure for the performance of real-time perception tasks, such as OD and SLAM, on unconstrained road scenarios, particularly on onboard hardware such as resource-constrained edge devices.

## 3. Architecture

### 3.1. Test Vehicle Architecture

The developed test vehicle infrastructure had to be able to provide a stable data exchange network between the various perception sensors and the final computation endpoints that would execute object detection and SLAM tasks, as well as all the power needed and mechanical support for all of its components. The base car used was a five-door Mercedes Benz E Class Station Wagon (S213). The station wagon chassis type was selected especially due to its spacious trunk for interior equipment installation, and for its long low roof to accommodate all the sensors and required mechanical structure with easy access.

**Figure 1:**
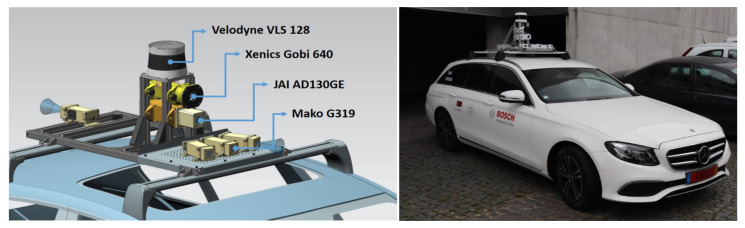
A 3D render of the test car sensor setup (**left**), Final setup installed on the test car (**right**).

The mechanical structure was built with extruded aluminum profiles and attached to a pair of roof bars with appropriately sized T slotted nuts. It is composed of a base frame that covers almost the entire roof, with a front-mounted perforated steel plate, and a center tower. The full setup comprises the following sensors and can be observed in Figure 1:

- 1× Velodyne VLS 128 (LiDAR);
- 1× Xenics Gobi 640 (Thermal Camera);
- 1× JAI AD130GE (NIR +RGB Camera);
- 4× Mako G319 (RGB Camera).

However, for the purpose of this article, the main sensor is the LiDAR; nonetheless, the test vehicle is equipped to act as a platform for both data collection, performance analysis, and practical test runs that enable the development and field validation of perception pipelines.

The LiDAR is mounted on top of the center tower with a clear 360° field of view of the road. The thermal and NIR+RGB cameras occupy the space in the center tower below the LiDAR. The RGB cameras are all placed on the front steel plate, covering the left, center, and right view, except for one RGB camera that is mounted at the rear directly onto the base frame in order to obtain a perspective of the rear view of the vehicle. The orientation of the RGB cameras is easily adjustable if needed. All the cameras are housed in ventilated 3D-printed covers. These covers are designed to avoid accidental damage caused by rain and road debris. On each side of the base frame, there is a watertight junction box where all the required wiring looms meet. One side accommodates the data-related cables and the other, the power supply connections. To power all the installed equipment, a cable runs from the roof structure to the car backseat through the left back door and connects to a power control box. This power control box has switches to turn each sensor on and off individually and connects to a 12-volts 15-amps socket.

All the selected sensors are equipped with 1Gbps ethernet interfaces. To connect all the sensors in a network and guarantee that there is enough bandwidth to capture data from all the sensors reliably, the usual approach would be to use a powerful enough network switch and create a conventional LAN with a start topology. However, this presents the following two problems: powerful switches are usually big enough and require a considerable amount of power that its installation in a vehicle is complicated, and the bandwidth that can be processed is ultimately capped by the bandwidth of the network card of the processing computer. The computer to be used was a Fujitsu CELSIUS H770 and it is equipped with a 1Gbps ethernet interface. To solve these problems, instead of a network switch, the network was created using a Thunderbolt 3 hub connected to the Thunderbolt 3 enabled USB-C port of the laptop. The TS3-Plus hub from CalDigit was used and enclosed in one of the watertight junction boxes. This hub has eight extra USB ports as follows:

- 5× USB-A 3.1 (Gen. 1 5 Gbps);
- 1× USB-C 3.1 (Gen. 1 5 Gbps);
- 1× USB-C 3.1 (Gen. 2 10 Gbps);
- 1× USB-C Thunderbolt 3 (40 Gbps).

To each one of these extra ports, a USB ethernet 1Gps network card was attached, connecting to each sensor. This setup allows the use of the 40 Gbps Thunderbolt 3 port of the laptop to connect each sensor of the setup, greatly increasing the available bandwidth for data exchange, at a cost of not having other useful features from a managed switch-like, sensor interconnectivity, VLAN control, load management, among others. The data processing is performed under ROS. Broadly speaking, each sensor is represented by a ROS nodelet running in the processing laptop that captures the streamed data and redirects it to the main ROS core process. Each recording nodelet captures all the relevant data and stores it, if necessary, to .rosbag files. In the case of the LiDAR sensor, network packets are streamed from the sensor and picked up by a special ROS nodelet, which converts the packets into usable 3D point cloud points. These point cloud points are then broadcasted back to the ROS core process and are available for use for tasks such as object detection or SLAM.

### 3.2. Object Detection Algorithm

**Figure 2:**
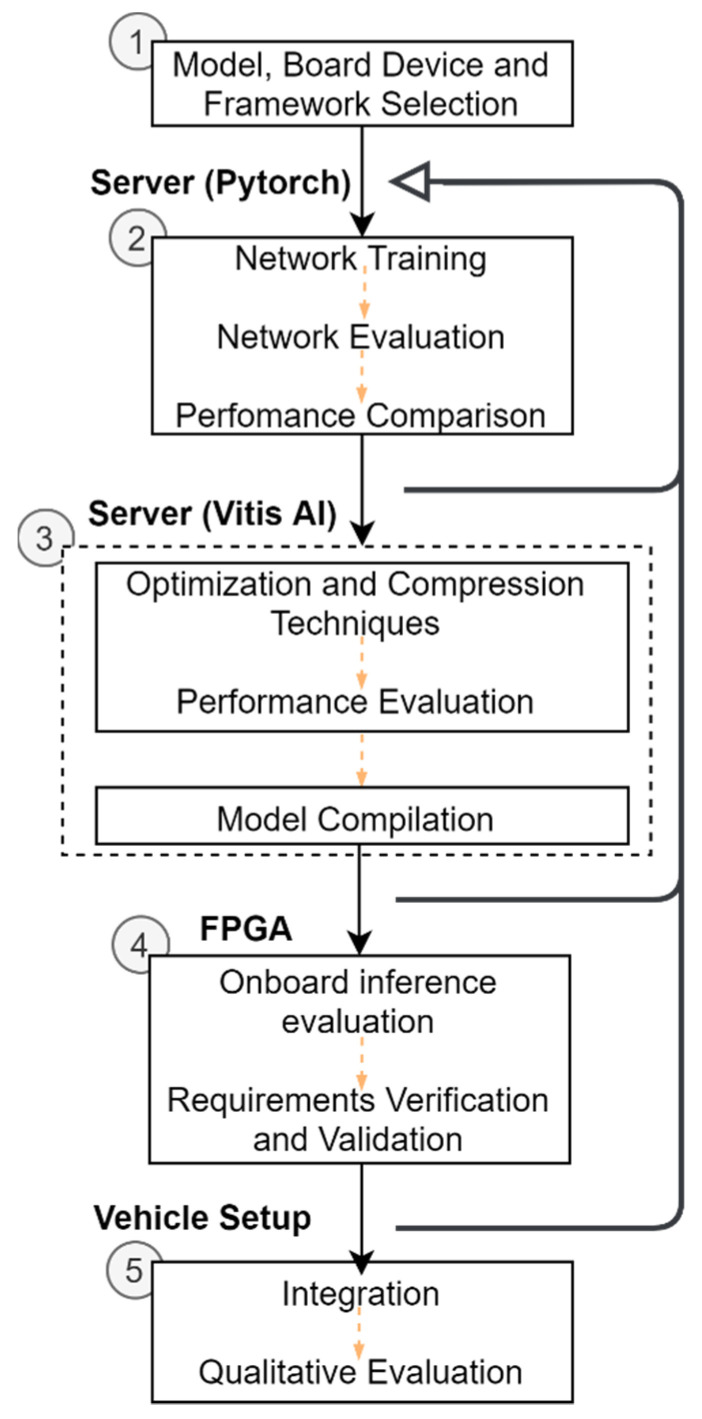
The methodology proposed for implementing an onboard inference in a vehicle setup equipped with a LiDAR.

Figure 2 depicts the five-step methodology followed to implement the deep learning-based inference model in the vehicle setup. It includes a literature review for selecting a 3D Object Detection model, an FPGA-based platform (Xilinx Ultrascale + ZCU102) and a compatible framework (Vitis AI). After this step, the 3D object model is subjected to the training and evaluation phase, both performed on the server-side (Intel Core i9 with 64 GB RAM and a Quadro RTX 6000 GPU) to ensure that our model meets the application requirements on the server. In this context, the proposed development flow follows an iterative approach, where the model is updated, and the steps of training and evaluation are repeated whenever required. The KITTI dataset [15] is used for the evaluation of this workflow. When this goal is accomplished, Xilinx tools are used in the deployment phase (3). Here, Vivado and Petalinux are used to configure and build hardware and software components for the selected board. Vitis AI allows the optimization and compression of the model obtained in the previous step. Before mapping model’ operators to instructions for the Intellectual Property (IP) core, called Xilinx Deep Learning Processing Unit (DPU), provided by Xilinx, the performance of the optimized and compressed version of the 3D object detection model is analyzed with regard to accuracy degradation and speed improvement. The process flow only starts the compilation step (3) when an optimal balance between performance metrics is achieved. Otherwise, the development flow restarts in stage (2). As steps in (3) are performed in the server, it only provides insights about the accuracy and trade-off between metrics, the condition criteria for an on-board inference time lower than 100 ms needs further validation. To do so, Vitis AI output files are loaded onto the FPGA-based board for model execution and the inference times are observed. To assess inference time criteria, but with satisfactory accuracy performance, steps (2), (3), and (4) might need to be repeated. A detailed description of the platform generation flow is provided in [35].

For the selection of a 3D Object Detection model, the main criteria used were of a low complexity and had a good balance between performance metrics. We opted for the model PointPillars, as this model relies on 2D dense convolutions to the detriment of more complex 3D convolution-based features extraction networks and achieves high-quality detection within short intervals of time. To make this possible, in the pre-processing stage, the point cloud is restructured into a volumetric representation in the form of pillars, and data augmentation is performed on each pillar for each of its points, up to a maximum of 100 per pillar, leading to points holding nine features. These features are then subjected to a linear layer, called the Pillar Feature Network, resulting in a set of 64 features for describing the geometrical and location information per pillar. Computed features are scattered back to the pillars’ original positions, generating a 64 channel pseudo-image, which enables the application of the 2D CNNs-based RPN for feature extraction. Finally, a lighter version of the Single Shot Detector network [36] is used as a detection head. PointPillars uses a feature extraction network that contains three sequential convolution blocks composed of 3, 5, and 5 convolution layers and 64, 128, and 256 filters, respectively.

### 3.3. SLAM

**Figure 3:**
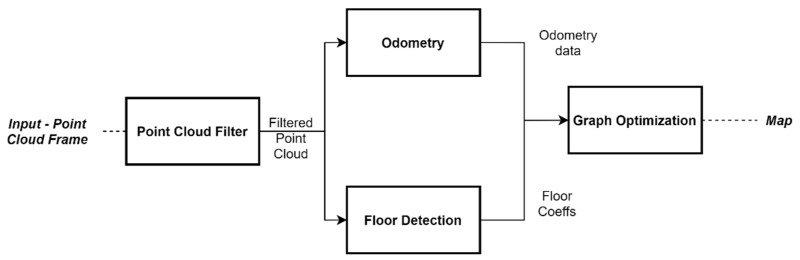
Flow process for GraphSLAM task.

In this work, we propose an architecture for GraphSLAM using the open-source framework ROS [37]. Our proposal is aimed mainly at 3D LiDAR data assembled on cars. In ROS, processes are represented as nodes in a graph structure, connected by topics, which is very convenient for GraphSLAM task. Figure 3 shows the processes flow of the proposed GraphSLAM, where four main processes stand out, namely, Point cloud filter, Odometry, Floor Detection, and Graph Optimization.

The point cloud filter allows for the downsampling of the point cloud, i.e., reducing the number of points using a voxelized grid approach. Next, to perform the odometry related operations, we followed the approach presented in [24], where a multi-threaded GICP, running in a GPU, is addressed. This approach does also implement an NDT algorithm. Regarding the Floor detection process, it relies on the RANSAC algorithm to add edges of constraint. This approach is useful when no loop is detected in the trajectory.

**Figure 4:**
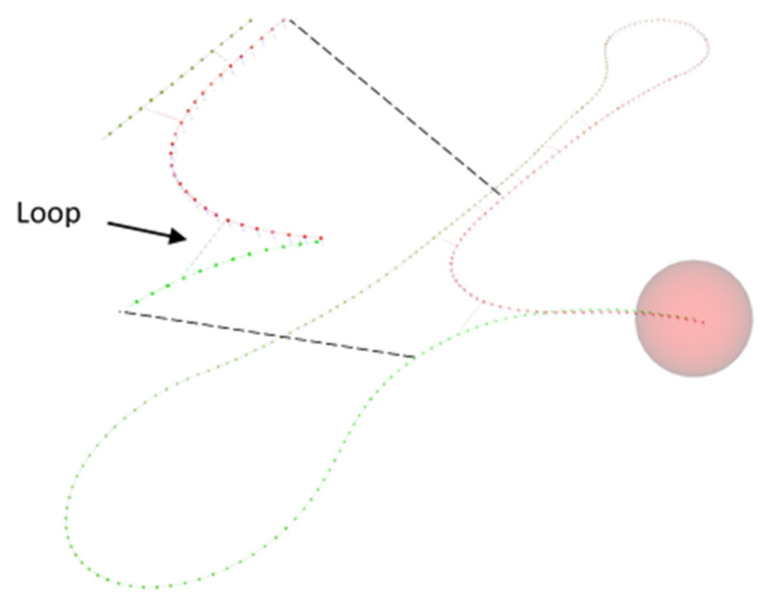
Loops close detections.

Due to the advantages discussed above, mainly in scalability and efficiency, we propose to use the g2o framework for graph optimization. However, according to the problem in the efficiency, due to the size increase with the trajectory, we opted to prune the resulting graph. This method is applied when there is at least a loop-closing. The detection of a loop-closing (Figure 4.) consists of recognizing whether the current position of the car was previously visited.

A graph was built by leveraging data outputted from the odometry process, where vertices correspond to vehicle poses labelled with information of the absolute pose (translation and rotation) concerning a fixed vertex (often, the start point of the car). Each edge contains the translation and rotation relative between its vertices and the uncertainty associated with the connection.

**Figure 5:**
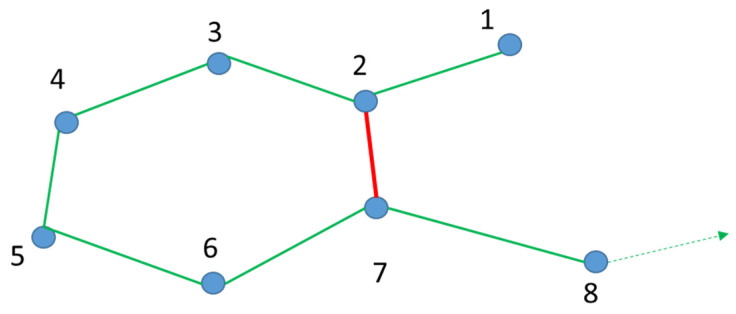
PoseGraph built from odometry data.

Taking Figure 5. as an example, we have a set of vertices *V* = {1,2,3,4,5,6,7,8}, a set of edges *E* = {12,23,27,34,45,56,67,78} and a loop-closing represented by the edge {27}, while paths are seen as a list of ordered vertices

In the example displayed in Figure 5, *P_t_* = {1,2,3,4,5,6,7,8} corresponds to the car trajectory. However, there is another path, *P*2 = {1,2,7}, which can be defined from vertex {1} to vertex {7}.

These graphs are updated here, i.e., vertexes and edges are added to the already existing graph every time the car reaches a new position. Finally, when the vehicle arrives at vertex {7}, a loop-close is performed (edge {27}) and added to the graph and ultimately path *P*2 is detected. The loop-closing adds important contradictory information in the graph that removes the drift error from the odometry process.

**Figure 6:**
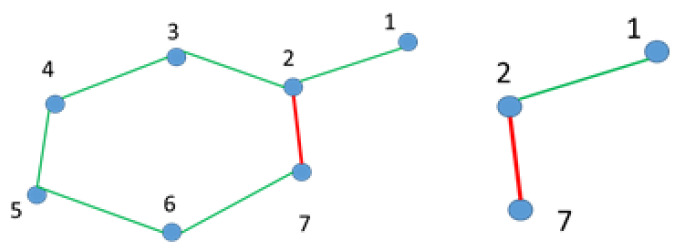
Graph before pruned (**left-hand side**) and pruned graph (**right-hand side**).

Graph Pruning is only applied right after the vehicle has reached vertex {7}, and the result of this operation is represented as follows: *G*{*V*, *E*}, where *V* = {1,2,3,4,5,6,7} and *E* = {12,23,27,34,45,56,67} (c.f. Figure 6. Left).

Let *V_rem_* = {3,4,5,6} be the difference between both paths, i.e., removing all elements in P1 present in P2 and *E_rem_* = {23,34,45,56} to identify all of the edges formed by vertices *V_rem_* present in *E*. Thereafter, a pruned graph *Gp* = {*V_p_*, *E_p_*}, with *V_p_* = {1,2,7} and *E_p_* = {12,27}, is computed by removing the vertexes *V_rem_* and the edges *E_rem_* in *G*, as showcased in Figure 6 (right-hand graph). Due to the advantages brought by the inclusion of the loop-closer function on SLAM as discussed before, we performed a general optimization to the graph whenever a new loop was included.

## 4. Results

### 4.1. Object Detection Performance Analysis

After performing the workflow steps (1) to (4) with the original PointPillars pipeline configuration, we could conclude that the quantized version of the original PointPillars configuration is viable for model inference in this application without having a substantial impact on performance when running it on the server-side, as shown in Table 2. However, when running on the edge device, this difference increases massively, almost doubling the inference time and failing to meet the inference time application requirement. It occurs due to the resource-constrained environment of the edge device.

The most compute-intensive pipeline stage is the feature extractor; thus, we have focused on updating this stage’s configuration. Upon performing the workflow in Figure 2. several times, we identified the following configuration, which keeps the number of blocks and layers but with fewer filters, namely, 32 (for layers in block one), 32 (block two), 64 (block three), as the most beneficial for on-board inference. Reducing the number of filters decreases the number of feature maps and channels and the number of arithmetic operations. As a result, the original feature extractor configuration achieves higher precision results over the selected configuration.

This trend is also observed for quantized models, c.f. Table 2, with the most complex structure achieving higher results. The proposed methodology and model updates led to positive results by surpassing the requirements by over 60% in inference speeds for both configurations.

Reducing the complexity of the feature extraction network has almost no effect on the model accuracy performance for the detection of large objects, such as cars, as suggested by the floating-point model results in Table 3. However, a significant score drop happens for cyclist detection. The effect of the compression techniques on performance follows the same trend, as quantized models still offer high-quality detection for cars and pedestrians. For instance, the score of the quantized sample of the original PointPillars is just around 0.5% lower than the floating-point model. However, adjusting the arithmetic operations for hardware limitations of the edge device, converting weights, bias, and input data for an 8-bit fixed-point representation leads to the scores dropping by around 10% for the detection of cyclists.

Therefore, compression techniques are superb in reducing the inference time while maintaining notable performance for most of the objects, but with a high cost on cyclist detections.

**Figure 7:**
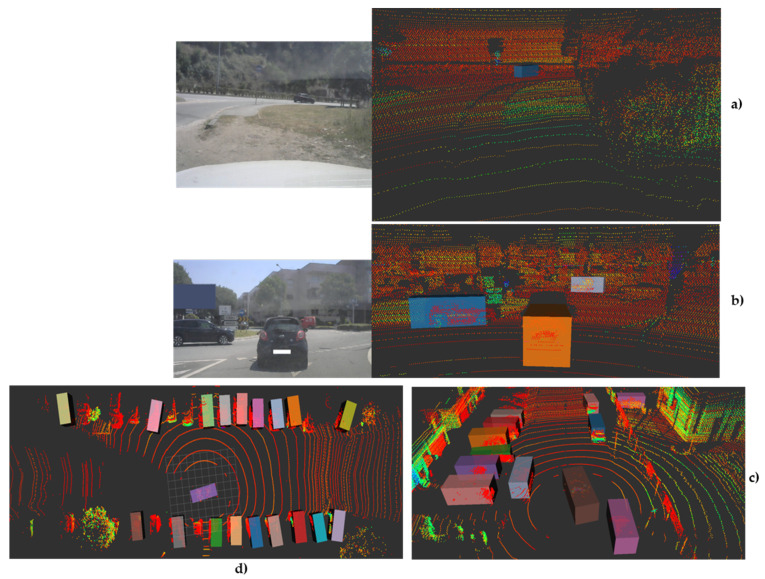
A 3D Object detection output on different scenarios: (**a**) test vehicle parked and dynamic objects on toad ahead; (**b**) Highlight of detected objects in typical approach to roundabout; (**c**) overtake scenario; and (**d**) Top-down view of a sample of moving test vehicle in a densely occupied area.

**Figure 8:**
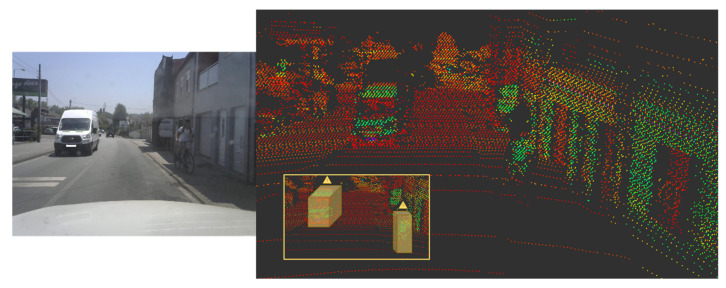
Driving test vehicle with van and cyclist in view.

As expected, these changes can be observed in the qualitative performance analysis. Figure 7 and Figure 8 show the performance of the proposed model for the abovementioned classes. Figure 7 shows some examples of the detection operation over different point clouds captured in different scenarios, where we can observe that the detection of the class Car is not significantly affected by the quantization application. This conclusion is also based on the results in the previous table. However, the same cannot be stated for small objects, as in frames containing such objects, the number of false negatives increases, as shown in Figure 8. Here, the proposed model did not detect the van as expected, since the model was not trained to detect this type of object, and thus it is not seen as a false negative but as a true negative, while the cyclist remained unseen in some frames during this sequence by the proposed model, while their floating counterpart model could detect it.

Energy consumption and resource usage are also two relevant performance metrics. Although GPU-based servers are the preferable hardware for running such computing demanding 3D object detection models, its application in vehicle setups is not reasonable due to power consumption and space demand. Although hybrid FPGA-CPU devices do not provide the same level of computation power, they demand less power supply while offering satisfactory flexibility. The hardware configuration of this platform directly affects power consumption, resource utilization, and throughput. In this context, the DPIU instantiated in the FPGA is configured with two cores, one for each of the model deep learning processes found in the PointPillars pipeline, i.e., Pillar Feature Network and RPN, while the remaining stages of its pipeline, which refer to the pre-processing and post-processing stages, are performed in the CPU side of the hardware platform.

This configuration along with the maximum parallelism provided by the target device achieves power consumptions lower than 15 W, while consuming just 60% of the total BRAM blocks and DSP units available, while the Slice LUTs and CLB registers usages is lower than 30%. The inference time performance achieved in this work provides a margin (40 ms) to reduce the power consumption and resource usage, while the solution keeps meeting the inference time requirement. For instance, updating DPU for using a single core reduces the power consumption by 5 W and resource usage by 30%, while a penalty of 10 ms is reported. This is a relevant aspect, as it reduces the power consumption for levels that can be easily supported by the test vehicle, as more than 50% of the resources in the FPGA-side are still available for the implementation of other algorithms, such as the SLAM.

### 4.2. SLAM Performance Analysis

The data acquired by the setup vehicles are used to assess the performance of the proposed solution regarding metrics efficiency and accuracy. For this purpose, we assess two methodologies, the pruned and unpruned graphs.

**Figure 9:**
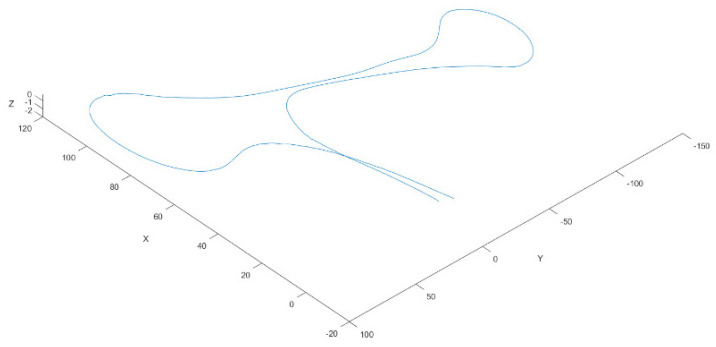
Vehicle trajectory obtained by the odometry process for a downsampling resolution of 0.5.

**Figure 10:**
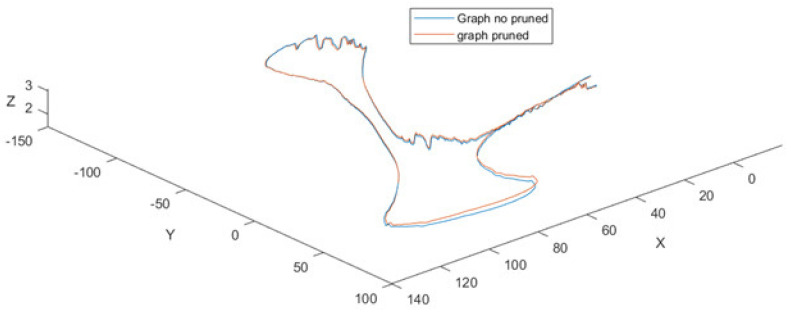
GraphSlam (blue) and GraphSlam subject to pruning (red).

Figure 9 illustrates a trajectory, with a duration of 123 s, at a sampling rate of 10 Hz frames/point clouds that composes this trajectory containing, on average, 170,000 points. Figure 10 shows the effect of accumulated error drift for trajectories that end at the start point. The efficiency of the odometry process to this trajectory is 0.01 s, and the drift error is near to 11.1 m.

Figure 10 shows the trajectory of the previous figure but with and without the application of the graph pruning. The results of its application can be seen in Table 4. The reported results show an improvement in the efficiency of about over 9.5 s in the graph optimization process between pruned and not pruned graphs. According to Figure 3, the rest of the following processes: Filtered points, Floor detection, and Odometry, consume nearly 16.2 s. The time elapsed when the no-pruned graph is applied is 84.5 s, while the pruned graph process takes 75 s.

On the other hand, the distance between the endpoints of the trajectories (0.39 m) is less than the maximum error of the trajectory (0.53 m), which shows there is not a continuous increase in the error when the pruned graph strategy is used (Figure 9.).

**Figure 11:**
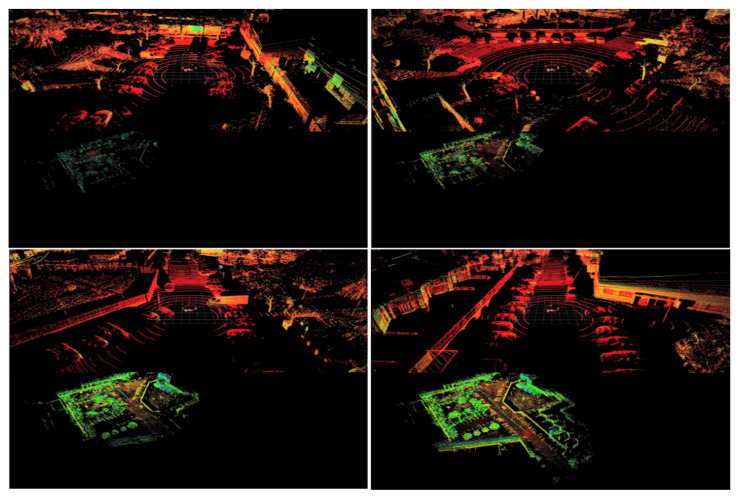
Examples of captured point cloud data and location and mapping data for a sequence of frames, where the image from the top corner is the first extracted frame and the bottom right consists of the last frame of this sequence. Every image displayed here is vertically divided into (**top**) point cloud provided by the LiDAR sensors and (**bottom**) SLAM output.

**Figure 12:**
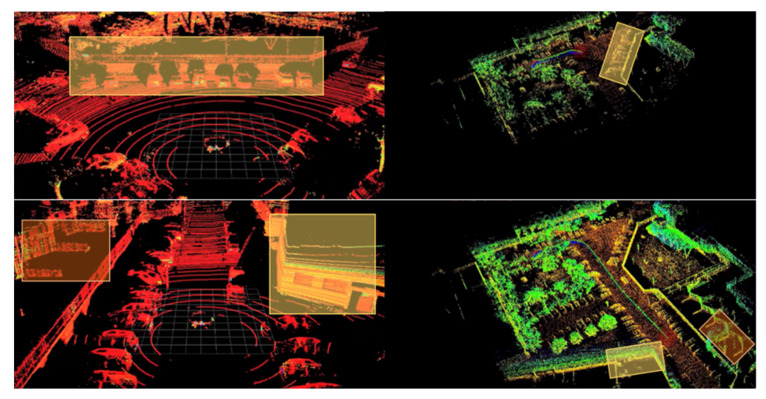
A highlight of identifiable world features in point cloud data (e.g., well-defined walls and parked cars) and corresponding data in the constructed map.

To better demonstrate some of the obtained location and mapping results from the prototype, we start by showing a short sequence where the test vehicle starts in a parking lot, takes a right-hand turn and drives through a straight main road. This is depicted in Figure 11 and Figure 12, corresponding to the first and last moments of data capture and processing, and to the identification of world features in the point cloud and corresponding data in the constructed map, respectively. Both Figures are output of the processing of the same point cloud sequence, but Figure 11 displays the trajectory whereas Figure 12 shows how the mapping generation is performed. 

In the above figure, the estimated locations for the test vehicle are represented by the colored circles leaving a trail through all the visited locations, with the current estimate represented by a big red translucent circle. In addition, the point cloud in Figure 11 is not the point cloud returned by the LiDAR sensor, but rather the estimated map points produced by the prototype. To enable this comparison, the following set of figures (through) show several examples of the current captured point cloud (top) and the estimated map points and locations (bottom) for the same sequence.

With the presented sequence, it is possible to see how the map-building process evolves and how the surrounding world features are accurately updated in the map. Figure 11 shows some examples of it. Here, in the top left corner, the vehicle is parked and then sets off, moving through a road. In the last extracted frame, we can see a line describing the trajectory of the vehicle since we left its parking spot.

**Figure 13:**
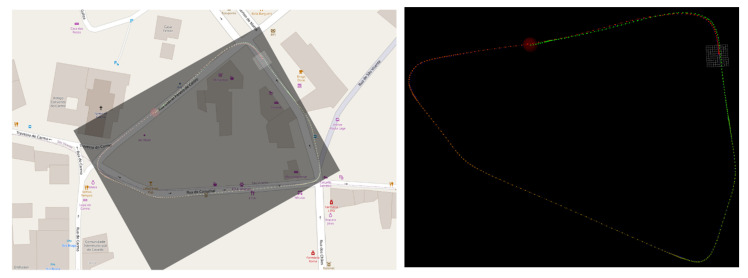
Example of a driving sequence: (**left**) Manual overlap of map view with computed vehicle positions in the map; and (**right**) Driving sequence featuring the loop, displaying only the estimated vehicle positions.

Test vehicle positions naturally evolve during a driving sequence. In some situations, rather than just constantly driving in new environments, some of the courses will feature a loop (i.e., a sequence where, during the drive, the vehicle returns to a previously visited location). In the figure presented below, such a case is demonstrated, with a snippet of a map view of the traversed area on OpenStreetMaps, a computed map and their overlap in Figure 13.

**Figure 14:**
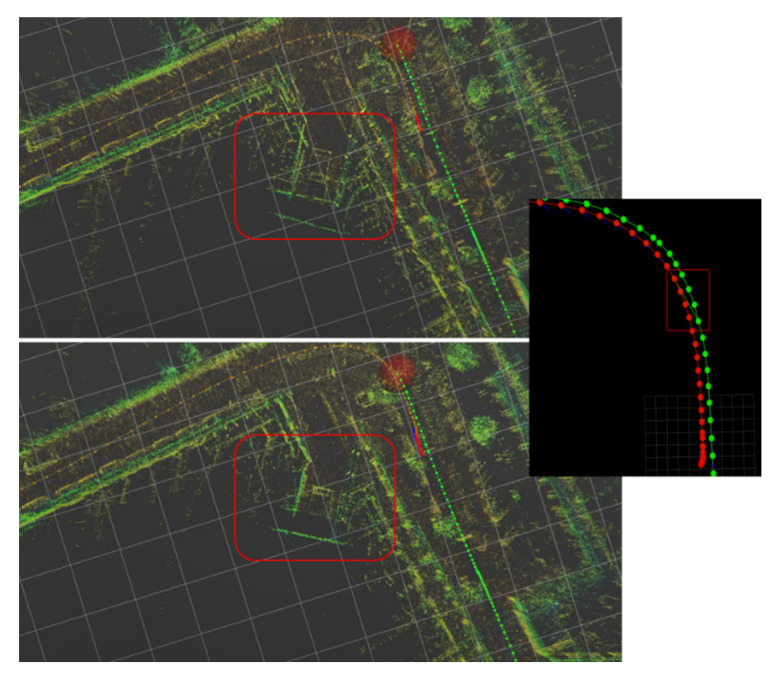
Map points and vehicle positions (**top**) prior and (**bottom**) after closer loop detection, with the highlight of a map closure event occurring. The green line corresponds to the first trajectory, whereas the red line represents the trajectory of the second time that the vehicle passes through the same place.

Additionally, Figure 14 shows the moments before and after the loop in that sequence being detected and the update of the map points.

## 5. Conclusions

This research work focused on addressing a low-cost test vehicle setup able to integrate both sensors and processing hardware for running important algorithms for efficient autonomous vehicles, as is the case of 3D Object detection and SLAM.

The main goal of the research works on 3D Object detection it is to enhance the precision performance on the server-side, where no considerations are taken about the requirements and restrictions of the real-case applications. The adoption of devices smaller in size, but also power consumption, computation power, and memory resources has shown to require some adaptions to the 3D Object Detection models. A methodology was proposed, covering the generation and configuration of both hardware and software, the 3D Object detection fine-tuning, and compression techniques’ application, aiming at achieving an optimal trade-off between precision and processing time, while the inference time requirements imposed by the application are met. The proposed hardware platform shows it to be able to assure such a goal, where the model performance matches with the server-side version for large objects such as cars, while degradation of up to 10% was reported for small objects, such as cyclists. Moreover, the average inference time is 60 ms, quite a lot lower than the inference time threshold (100 ms). Regarding the model accuracy, it was probed to perform properly on the task of detecting vehicles; however, the accuracy drops significantly for small objects, such as vehicles and pedestrians. Given the application nature, it is mandatory for solutions to be robust and reliable. In this sense, our results have shown that due to the limitations of LiDAR perceiving small objects, as well as its limitations under some atmospheric conditions, the 3D object detection solutions are not mature enough to fulfil all the application requirements. Therefore, a multi-modal approach seems to be the proper roadmap, where different sensors will complement each other, helping 3D object detection to overcome most of the limitations herein discussed.

The implementation solution for performing the role of SLAM in the test vehicle follows an architecture comprising four components, point cloud filtering, odometry, floor detection, and graph optimization. The latter component prunes the outputted graph, significantly improving the mapping and location tasks of the proposed approach. Moreover, the proposed solution has been shown to be capable of performing well in scenarios where the vehicle returns to a previously visited location, thanks to the loop-closure detection feature. However, long driving without returning to a previously visited location affects the ability of the solution to compute the vehicle location due to error drifts. Therefore, this error might increase to unacceptable values after several hours of vehicle utilization. This limitation shows that it might be required for LiDAR to be fused with other sensors, for instance, GPS or IMUs, to mitigate this error location accumulative error.

This paper addresses the steps required to integrate both the developed OD and SLAM prototypes on the test vehicle. Afterward, we presented the results obtained by feeding the prototypes with live data from a Velodyne VLS-128 LiDAR sensor on test drives and provided a qualitative evaluation of the obtained results for each of the prototypes. The results demonstrate the applicability of the developed prototypes to perform soft real-time OD and SLAM on live data from the test vehicle.

## Figures and Tables

**Table 1:** Platforms and sensor setups.

Vehicle	Year	Sensor Setup	Ref.
Volkswagen Passat	2008	5 LiDARs (different manufacturers), 5 RADARs, GPS-based Inertial Navigation System (INS)	[30]
Chevrolet Tahoe	2008	10 LiDARs, 3 RADARs (2 front, 1 rear), 2 cameras	[29]
Volkswagen Passat B6	2012	4 Cameras (2 grayscale, 2 color), LiDAR, OXTS positioning system	[3]
Mercedes Benz S class	2013	Stereo camera (front), 2 mono cameras (front/back), 4 short-range RADARs, 4 long-range RADARs	[31]
Different vehicles	2017	Short-, mid-, and long-range LiDARs, camera, RADAR, GPS; also features audio detection	[32]
Renault Zoe	2019	Cameras (color), LiDAR, 5 RADARs	[33]

**Table 2:** Inference Time, given in ms, metric benchmark results.

Model Configuration.	Floating-Point Model	Quantized Model (Server)	Quantized Model (Edge Device)
Original PointPillars Conf.	47.0	42.6	105.3
Configuration updated for resource constraint devices	43.0	38.4	60.3

**Table 3:** Accuracy Degradation (denoted as the difference in performance from the original configuration to the new configuration) results on KITTI BEV.

Model Configuration		Floating-Point Model (%)			Quantized Model (%)		
		Easy	Moderate	Hard	Easy	Moderate	Hard
Original PointPillars	Car	N.A	N.A	N.A	−0.2	−0.68	−0.38
	Cyclist	N.A	N.A	N.A	−5.3	−6.9	−3.3
	Pedestrian	N.A	N.A	M.A	−3.8	−7.2	−2.6
Configuration updated for resource constraint devices	Car	0.22	0.44	0.27	−0.46	−8.2	−1.18
	Cyclist	−7.31	−8.92	−5.46	−8.79	−10.85	−10.59
	Pedestrian	−1.53	−5.62	−6	−2.71	−6.96	−1.48

**Table 4:** Performance between no-pruned and pruned graphs. In both cases, the detected loop count is equal to 11 and the distance between the starting and endpoints is equal to 0.39 m.

Pruned Graphs	Statistic of the Correspondence between Trajectories	Total Time of Graph-Optimization in the Trajectory (s)
No	*mean_dis_* = 0.32 m *max_dis_* = 0.53 m *std_dis_* = 0.1 m *var_dis_* = 0.01 m	68.3
Yes		58.8
